# Supplementary material for: Conditional chemoconnectomics (cCCTomics) as a strategy for efficient and conditional targeting of chemical transmission
Source: eLife. 2024 Apr 30;12:RP91927. doi: 10.7554/eLife.91927 (PMC11060718; doi:10.7554/eLife.91927)
Supplement: Figure 3—source data 3. [file elife-91927-fig3-data3.zip › Figure 3 source data3 part 1.zip!/Figure 3-Source data 3-part1/1175-9-CRISPResso_Report_rtnd62ko.zip!/CRISPResso_on_1175_12/1a.Read_barplot.pdf]

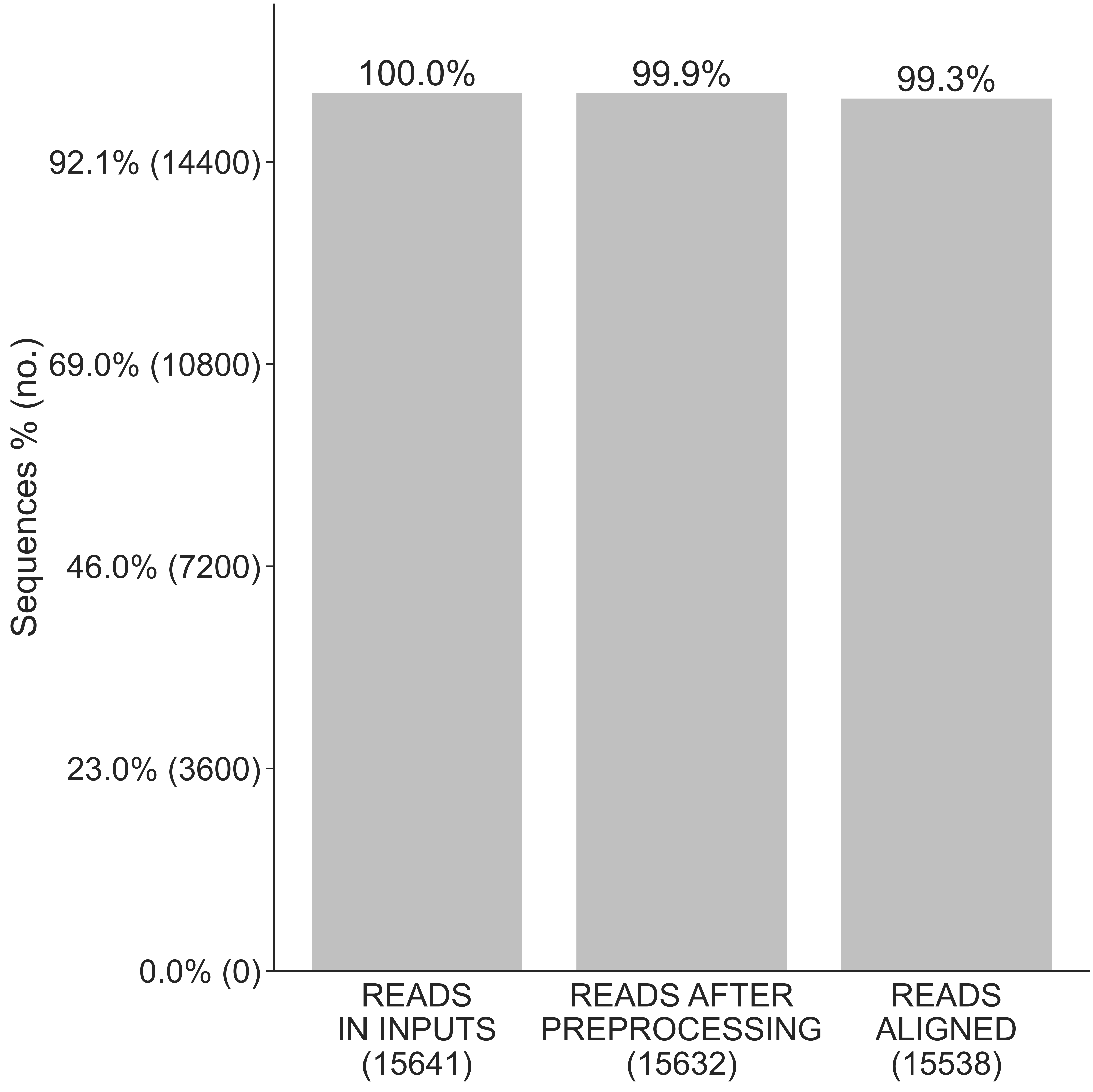

Sequences % (no.)
92.1% (14400)
69.0% (10800)
46.0% (7200)
23.0% (3600)
0.0% (0)
100.0%
99.9%
99.3%
READS IN INPUTS (15641)
READS AFTER PREPROCESSING (15632)
READS ALIGNED (15538)
